# Brain capillary obstruction during neurotoxicity in a mouse model of anti-CD19 chimeric antigen receptor T-cell therapy

**DOI:** 10.1093/braincomms/fcab309

**Published:** 2021-12-31

**Authors:** Lila D. Faulhaber, Anthea Q. Phuong, Kendra Jae Hartsuyker, Yeheun Cho, Katie K. Mand, Stuart D. Harper, Aaron K. Olson, Gwenn A. Garden, Andy Y. Shih, Juliane Gust

**Affiliations:** Center for Developmental Biology and Regenerative Medicine, Seattle Children’s Research Institute, Seattle, WA 98101, USA; Center for Integrative Brain Research, Seattle Children’s Research Institute, Seattle, WA 98101, USA; Center for Integrative Brain Research, Seattle Children’s Research Institute, Seattle, WA 98101, USA; Center for Integrative Brain Research, Seattle Children’s Research Institute, Seattle, WA 98101, USA; Center for Integrative Brain Research, Seattle Children’s Research Institute, Seattle, WA 98101, USA; Center for Integrative Brain Research, Seattle Children’s Research Institute, Seattle, WA 98101, USA; Center for Integrative Brain Research, Seattle Children’s Research Institute, Seattle, WA 98101, USA; Department of Pediatrics, University of Washington, Seattle, WA 98195, USA; Department of Neurology, University of Washington, Seattle, WA 98195, USA; Center for Developmental Biology and Regenerative Medicine, Seattle Children’s Research Institute, Seattle, WA 98101, USA; Department of Pediatrics, University of Washington, Seattle, WA 98195, USA; Department of Bioengineering, University of Washington, Seattle, WA 98195, USA; Center for Integrative Brain Research, Seattle Children’s Research Institute, Seattle, WA 98101, USA; Department of Neurology, University of Washington, Seattle, WA 98195, USA

**Keywords:** CD19 chimeric antigen receptor, neurotoxicity, neurovascular unit, capillary, two-photon imaging

## Abstract

Immunotherapy for haematologic malignancies with CD19-directed chimeric antigen receptor T cells has been highly successful at eradicating cancer but is associated with acute neurotoxicity in ∼40% of patients. This neurotoxicity correlates with systemic cytokine release syndrome, endothelial activation and disruption of endothelial integrity, but it remains unclear how these mechanisms interact and how they lead to neurologic dysfunction. We hypothesized that dysfunction of the neurovascular unit is a key step in the development of neurotoxicity. To recapitulate the interaction of the intact immune system with the blood–brain barrier, we first developed an immunocompetent mouse model of chimeric antigen receptor T-cell treatment-associated neurotoxicity. We treated wild-type mice with cyclophosphamide lymphodepletion followed by escalating doses of murine CD19-directed chimeric antigen receptor T cells. Within 3–5 days after chimeric antigen receptor T-cell infusion, these mice developed systemic cytokine release and abnormal behaviour as measured by daily neurologic screening exams and open-field testing. Histologic examination revealed widespread brain haemorrhages, diffuse extravascular immunoglobulin deposition, loss of capillary pericyte coverage and increased prevalence of string capillaries. To measure any associated changes in cerebral microvascular blood flow, we performed *in vivo* two-photon imaging through thinned-skull cranial windows. Unexpectedly, we found that 11.9% of cortical capillaries were plugged by Day 6 after chimeric antigen receptor T-cell treatment, compared to 1.1% in controls treated with mock transduced T cells. The capillary plugs comprised CD45+ leucocytes, a subset of which were CD3+ T cells. Plugging of this severity is expected to compromise cerebral perfusion. Indeed, we found widely distributed patchy hypoxia by hypoxyprobe immunolabelling. Increased serum levels of soluble ICAM-1 and VCAM-1 support a putative mechanism of increased leucocyte–endothelial adhesion. These data reveal that brain capillary obstruction may cause sufficient microvascular compromise to explain the clinical phenotype of chimeric antigen receptor T-cell neurotoxicity. The translational impact of this finding is strengthened by the fact that our mouse model closely approximates the kinetics and histologic findings of the chimeric antigen receptor T-cell neurotoxicity syndrome seen in human patients. This new link between systemic immune activation and neurovascular unit injury may be amenable to therapeutic intervention.

## Teaser

In a mouse model of neurotoxicity in chimeric antigen receptor T-cell cancer therapy, Faulhaber *et al*. use *in vivo* two-photon imaging to show that over 10% of brain cortical capillaries become obstructed by leucocytes. This, accompanied by acute behaviour changes, suggests a possible new mechanism for immunotherapy-associated neurologic toxicity.

## Graphical Abstract

**Graphical Abstract:**
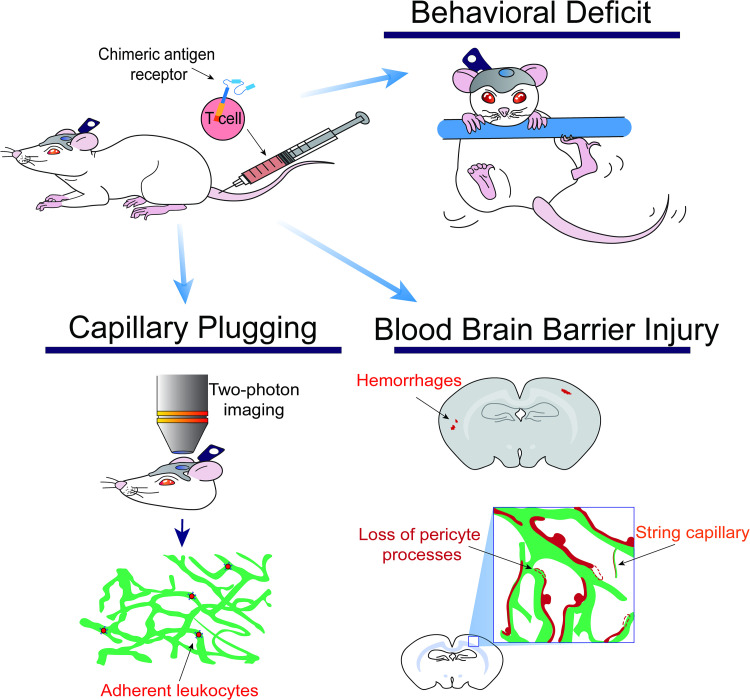

## Introduction

Chimeric antigen receptor (CAR) T-cell therapy has revolutionized the treatment of haematologic malignancies, but systemic and neurologic toxicities remain a major concern.^1^ CAR T cells work by recognizing surface targets on tumour cells via a chimeric receptor that consists of an extracellular binder, typically an antibody fragment, fused to intracellular signalling domains.^2^ Once the CAR T cells are infused into the patient, they recognize and lyse the target cells via the formation of a non-classical immune synapse.^3^ Binding of targets also induces CAR T-cell proliferation and systemic cytokine release. The greatest success of this therapeutic approach is seen in CD19^+^ haematologic malignancies such as acute lymphocytic leukaemia and non-Hodgkin’s lymphoma, with remission rates above 90% and worldwide regulatory agency approval of several CD19-targeting CAR T products.^4,5^

Despite these successes, neurologic toxicity remains a major concern in CAR T-cell therapy. Neurotoxicity, also termed immune effector cell-associated neurotoxicity (ICANS)^6^ occurs in ∼40% of patients receiving CD19-directed CAR T cells and is also seen after CD22- and B-cell maturation antigen-CAR treatment.^7–9^ We should note that in the oncology field, the term ‘neurotoxicity’ is used as a clinical descriptor of neurologic adverse events following cancer treatment, and is not meant to specifically imply neuronal cellular injury, though it is a facet of the injury. The most common symptoms are transient language disturbance or delirium, which are almost always accompanied by electroencephalogram background slowing.^10–13^ Approximately 10–20% of patients have potentially life-threatening manifestations such as seizures and coma. In some cases, neuronal injury is apparent by ictal and interictal encephalography patterns.^13–15^ MRI may show cortical diffusion restriction evolving into cortical laminar necrosis, consistent with the severe neuronal injury.^10,11^ Empiric treatments for ICANS include corticosteroids and interleukin (IL)-1 blockade, which is currently being evaluated in multiple clinical trials.^16^ Unfortunately, ∼1% of patients treated with CD19-directed CAR T cells develop rapidly progressive cerebral oedema that can lead to death despite aggressive immunomodulation and intracranial pressure-directed interventions.^7^ Autopsy findings in patients with cerebral oedema include microhaemorrhages, microvascular disruption and perivascular plasma extravasation, perivascular infiltration of T cells and macrophages, leptomeningeal accumulation of CAR T cells and astrocyte activation.^8,17^ The loss of neurons or demyelination has not been demonstrated in these cases. The mechanism of neurotoxicity remains incompletely understood. The most consistent clinical risk factor is the severity of cytokine release syndrome (CRS), which in turn is influenced by disease burden and amount of CAR T-cell proliferation.^16,18–20^ Dysfunction of the neurovascular unit likely plays a role, as evidenced by reversible interstitial oedema on MRI in some patients,^10–12^ and a shift of the angiopoietin–Tie2 axis towards endothelial activation that was demonstrated in several clinical studies.^10–12^

The most faithful animal model to date has been in non-human primates, which develop CRS and neurotoxicity after treatment with CD20-directed CAR T cells.^21^ In mice, no model has yet been described that recapitulates multiple key features of neurotoxicity, including time course, behavioural changes and/or histopathologic findings similar to those in humans.^8^ In immunodeficient mice treated with human CAR T cells, some aspects of neurotoxicity have been modelled and linked to systemic cytokine release. In a humanized mouse model of CD19-CAR T toxicity, the blockade of IL-1 prevented neurologic deterioration 4 weeks after CAR T-cell infusion.^22^ In a xenograft mouse model, the blockade of granulocyte-macrophage colony-stimulating factor reduced contrast extravasation on brain MRI.^23^ Most syngeneic mouse models, where murine CAR T cells are given to immunocompetent mice, have shown excellent CAR T-cell function but no toxicity.^24–28^ In the syngeneic studies that did demonstrate toxicity, detailed neurologic phenotyping has not yet been reported.^29–31^ We have developed an immunocompetent mouse model that develops CRS and neurotoxicity after high-dose cyclophosphamide lymphodepletion and high-dose second-generation murine CD19-directed CAR T cells. We show evidence of blood–brain barrier disruption, pericyte injury and capillary regression. By *in vivo* two-photon imaging of the cerebral cortex, we found persistent capillary plugging by leucocytes and tissue hypoxia, which supports a key role for microvascular dysfunction in the pathophysiology of neurotoxicity.

## Materials and methods

### Animals

Five- to 10-week-old wild-type BALB/c mice were used for all experiments. Male and female mice were used in equal numbers. Mice were obtained from Jackson Labs (Bar Harbor, ME, USA) and housed in specific pathogen-free facilities. After CAR T-cell treatment, ear temperature and weight were recorded daily and mice received 10 ml/kg normal saline subcutaneously daily. No animals were excluded from analysis, except for those which received test doses from CAR T-cell batches, which were deemed unsuccessful production runs. All experiments were approved by the Seattle Children’s Research Institute Animal Use and Care Committee.

### CAR T-cell generation

The CAR construct (gift from Juno Therapeutics) consists of an anti-murine-CD19 single-chain variable fragment derived from the 1D3 hybridoma^25^ and murine intracellular 4-1BB and CD3z costimulatory domains, followed by a T2A sequence to coexpress the transduction marker Thy1.1. The construct was cloned into an MMLV backbone and gammaretrovirus was produced by transient calcium phosphate transfection of a Phoenix-ECO producer line (ATCC). CAR T cells were generated per published protocol.^32^ Briefly, T cells were collected by mechanically dissociating spleens and lymph nodes of wild-type BALB/c mice, followed by MACS selection with CD90.2 beads (Miltenyi Biotec). T cells were then stimulated with CD3/CD28 Dynabeads (ThermoFisher) (Days 1–4), murine IL-2 (Peprotech) (Days 1–3) and murine IL-15 (Peprotech) (Day 4) in Roswell Park Memorial Institute (RPMI) 1640 medium supplemented with 10% foetal bovine serum, penicillin/streptomycin, β-mercaptoethanol and sodium pyruvate. On Days 2 and 3, cells were spinoculated with viral supernatant at 2000 × *g* for 1 h on retronectin (Takara) coated plates. Transduction efficiency was verified by flow cytometry for Thy1.1. Mock transduced T cells underwent the same transduction protocol, while replacing the viral supernatant with media during the spinoculation step. Each CAR T-cell batch was tested for efficacy by injecting three mice with 10 × 10^6^ CAR T cells. A batch was only used for further experiments if the test mice showed CAR T-cell expansion and B-cell depletion.

### CAR T-cell treatment

To reduce the rejection of CAR T cells, mice received lymphodepleting chemotherapy with 250 mg/kg cyclophosphamide via a single i.p. injection (Day 1). This cyclophosphamide dose was previously established as effective for lymphodepletion in immunocompetent mice.^30,33^ Twenty-four hours later (Day 0), 5–10 × 10^6^ CAR T cells or mock transduced T cells per animal were infused via the lateral tail vein. Mice were monitored via daily weight and temperature measurements and both controls and CAR T-treated animals received daily subcutaneous normal saline or lactated Ringer’s solution boluses (20 ml/kg). Blood for analysis was collected via chin bleed, retroorbital puncture under sedation or post-euthanasia.

### Flow cytometry

Spleens were removed and mechanically dissociated through a 70 µM filter. Blood was collected in EDTA tubes, RBCs lysed and lymphocytes collected via density gradient centrifugation in lymphocyte separation media. Mice were then perfused transcardially with ice-cold PBS to remove intravascular blood, and brains were dissected out. To recover brain-infiltrating leucocytes, we used mechanical homogenization and density gradient centrifugation.^34,35^ Brains were homogenized in RPMI 1640 medium in a 5 ml Dounce homogenizer and strained through a 70 µm filter. Cells were suspended in 30% percoll in RPMI 1640 medium and layered over a 70% percoll solution, followed by spinning at 500×*g* for 30 min at room temperature without brake. The myelin debris that collects at the top was removed and the 30/70% percoll interphase layer containing leucocytes was collected. Cells were then resuspended, labelled on ice with fluorescently conjugated antibodies against mouse CD45, CD3, CD19, Thy1.1 and viability dye (Biolegend), and analysed on an LSR Fortessa or Novocyte flow cytometer.

### Serum analysis for cytokines and adhesion molecules

Cytokine concentrations [CXCL1, interferon-γ (IFN-γ), IL-1β, IL-2, IL-4, IL-5, IL-6, IL-10, IL-12p70 and tumour necrosis factor (TNF)] in serum were measured using the V-PLEX Proinflammatory Panel 1 Mouse Kit (Mesoscale Discovery) according to the manufacturer’s instructions. For soluble adhesion molecule measurements, serum samples were diluted 1:100 and analysed per manufacturer’s instructions with the mouse ICAM-1/CD54 Quantikine ELISA kit and the mouse VCAM-1/CD106 Quantikine ELISA kit, both from R&D Systems.

### Histologic analysis

Fluorescently labelled dextrans (5%, 50 μl) (Thermo Fisher) were injected retroorbitally under isoflurane anaesthesia 30 min prior to euthanasia. Mice were euthanized with CO_2_ and transcardially perfused with ice-cold PBS followed by 4% paraformaldehyde (PFA). Brains were fixed in 4% PFA overnight, cryoprotected in 15 and 30% sucrose and cut into 20–50 μm coronal sections. For haemorrhage counts, we analysed H&E-stained 30 μm sections, which were taken every 1 mm from the rostral brain excluding the olfactory bulbs to the caudal end of the cerebellum. The haemorrhages per volume analysed were then normalized to a total brain volume of 500 mm^3^.^36^

### Immunohistochemistry

Twenty to 30 μm slide-mounted or 50 μm floating (for microglia and pericyte analysis) PFA-fixed brain sections were incubated in blocking solution (10% donkey serum + 0.1% Triton X-100 in PBS) for 1 h, then at 4°C overnight with primary antibodies in 1% donkey serum + 0.1% Triton X-100 in PBS, washed in PBS, incubated with secondary antibodies for 2 h at room temperature, counterstained with DAPI and mounted in Fluoromount G (Southern Biotech). Primary antibodies: Claudin-5 (mouse, clone 4C3C2, ThermoFisher cat# 35-2500, 1:100), Iba1 (goat polyclonal, Abcam cat# ab5076, 1:1000), laminin (rabbit polyclonal, Novus Biologicals cat# NB300-144, 1:50) and CD13 (rat, clone 123H1, MBL cat# M101-3, 1:100). For IgG fluorescence quantification, 20 μm sections were incubated with fluorescently conjugated antibodies directed either against mouse IgG or the IgG of control species (rat, goat or rabbit) (all raised in donkey, Thermo Fisher) in PBS for 2 h.

### Hypoxyprobe labelling

Fresh pimonidazole hydrochloride (Hypoxyprobe, Inc.; HP3-100Kit) was prepared at 60 µg/ml in sterile-filtered PBS and injected at a dose of 60 mg/kg into the retroorbital vein under isoflurane anaesthesia. Mice were removed from anaesthesia and observed for 90 min to allow for pimonidazole adducts to form in any hypoxic tissue. Mice were then euthanized with CO_2_ and transcardially perfused with ice-cold saline followed by 4% PFA. Brains were processed as above into 50 µm floating sections, which were incubated overnight at room temperature with anti-pimonidazole antibody (Pab2627 rabbit antisera; 1:100; Hypoxyprobe, Inc.; HP3-100Kit) in PBS with 2% Triton X-100, 10% goat serum and 0.1% sodium azide. Secondary antibody labelling as above.

### Confocal imaging and analysis

Slides were imaged on a Zeiss LSM 710 confocal microscope at 20× to 63× magnification. All analysis was performed blinded to treatment conditions using the Fiji image processing package.^37^  *IgG deposition* was quantified as the median ratio of immunofluorescence brightness (arbitrary units) against mouse versus control IgG in three + non-consecutive brain sections per mouse. To normalize between experiments, measurements were expressed as per cent of mock-treated control. For *microglia* quantification, Iba1-positive cells were counted manually on at least three separate *z*-stacks from different cortical areas per animal. *Pericyte* coverage was expressed as the length percentage of laminin-labelled microvessels that overlapped with CD13+ pericyte processes.

### Neurophenotype scoring

An observer blinded to the treatment group conducted a daily 20-item neurophenotype exam. This was adapted from a screening exam for neurologic phenotypes in mutant mice.^38^ Scored items include grooming, piloerection, respirations, extremity perfusion, eye-opening, body posture, tail posture, spontaneous activity, visual orienting, walk on cage edge, whisker reflex, eye blink, ear reflex, startle reflex, balance on the rod, climb onto the rod, reach for target from the suspended position, upper extremity grip strength, postural adjustment upon cage rotation and unusual or stereotyped behaviours. Each item was scored 0 = normal, 1 = performed with limitations/mildly abnormal or 2 = unable to perform/severely abnormal, and the total daily score was determined by summing all items. Animals were habituated to the exam for 3 days in a row before any experimental manipulation was started.

### Open-field test

All mice were naïve to the test and were habituated to the behaviour room for 30 min prior to testing. Testing was conducted under dim light, and the test box was thoroughly cleaned between animals. Mice were introduced into a 20 cm × 20 cm × 20 cm box with a 10 cm × 10 cm centre area marked. Video was acquired and analysed with Noldus software. Time spent in the centre area and total distance travelled were quantified for the initial 5 min interval after introduction into the test box.

### Echocardiograms

Mice were sedated with 1% isoflurane with 21% oxygen delivered via nose cone at 1 l/min. ECG leads were placed for simultaneous ECG monitoring. Echocardiographic images were acquired on a Vevo 2100 machine using an MS400 transducer (VisualSonics Inc., Toronto, Canada). M-mode measurements at the midpapillary level of the left ventricle were performed to measure end-systolic and end-diastolic diameters and calculate stroke volume.^39^

### Cranial window preparation and anaesthesia

We created a polished and reinforced thinned-skull window over the somatosensory cortex as previously described.^40,41^ Briefly, anaesthesia was induced with 4% isoflurane and additional pain control provided with buprenorphine. Mice were maintained at 1–2% isoflurane for surgery. After removing the skin, the skull overlying the sensory cortex was thinned to translucency with a handheld drill. The area was covered with cyanoacrylate instant adhesive (Loctite 401, Henkel) and a coverslip, and a custom head mount was attached using dental cement (Metabond, Parkell). Mice were allowed to recover for 24 h prior to imaging. During imaging, mice were anaesthetized with 1.5% isoflurane. All antibodies and intravascular tracers were delivered by injection into the retroorbital sinus. The blood plasma was labelled with 2 MDa dextran conjugated to either FITC or Alexa 680 (5% in PBS, 20–50 μl per injection). For *in vivo* leucocyte labelling, we injected either 50 μl of freshly prepared Rhodamine 6G (0.1% in PBS) or monoclonal fluorescent-conjugated antibodies (rat anti-mouse CD3, clone 17A2; mouse anti-mouse CD45.2, clone 104; all from Biolegend and used at 0.4 mg/kg i.v.).

### *In vivo* two-photon imaging

Imaging was performed on a Bruker Investigator microscope with Prairie-View imaging software, coupled to a Spectra-Physics InSight X3 tunable laser (680–1300 nm) set to 800 nm excitation. Green, red and far-red fluorescence emission was collected through 525/70 nm, 595/50 nm and 660/40 bandpass filters, respectively. For the first imaging session at baseline, we collected an overview map of the entire window using a 4× (0.16 numerical aperture, NA) air objective (Olympus; UPLSAPO). The overview map was used to identify areas with an unobstructed view of the cortical capillaries and to allow repeat imaging of the same areas at subsequent imaging sessions. We then switched to a 20× (1.0 NA) water-immersion objective (Olympus; XLUMPLFLN) and collected at least three 150 μm deep image stacks per mouse. For antibody imaging, stacks were collected pre- and post-injection of antibody to control for any non-specific fluorescence in capillary plugs.

### Two-photon image analysis

All analysis was performed in ImageJ/Fiji.^42,43^ Every microvessel segment within an image stack was assigned a unique identifier using ImageJ’s multipoint function. Vessel labels were matched between baseline, Days 4 and 6 image stacks of the same areas. To count as non-flowing, a capillary had to have no movement of red blood cells on all image slices where it was observable. A typical capillary was observed for at least 6 s, the time it took to acquire five 1 μm cuts. We did not include brief capillary stalling in our counts, where the flow can be slowed for <5 s by large white blood cells squeezing through a capillary. We then used the VasoMetrics ImageJ plugin^44^ to measure the diameter of each vessel segment at the full width at half-maximum intensity. Segments of diameter over 7 μm were excluded from analysis as they were likely to represent arterioles or venules.

### Statistical analysis

All behaviour, image and echocardiogram measurements and analyses were performed blinded to the treatment group. Where appropriate, normality tests were performed prior to statistical tests. When normality was met, parametric analysis (*t*-test, one-way ANOVA with Holm–Sidak post-test to account for multiple comparisons, Pearson’s correlation) was performed and means were reported. Otherwise, non-parametric analyses (Mann–Whitney test, Spearman rank-sum correlation) were performed and the median values were reported. For longitudinal measurements, we used linear mixed-effects modelling. The specific test used is indicated in the text and/or figure legends. Differences were considered statistically significant at the 95% confidence level. All statistical analyses were performed in GraphPad Prism.

### Data availability

All original data will be made available by the authors upon reasonable request.

## Results

### CD19-CAR T-cell treatment induces CRS and neurotoxicity

**Figure 1:**
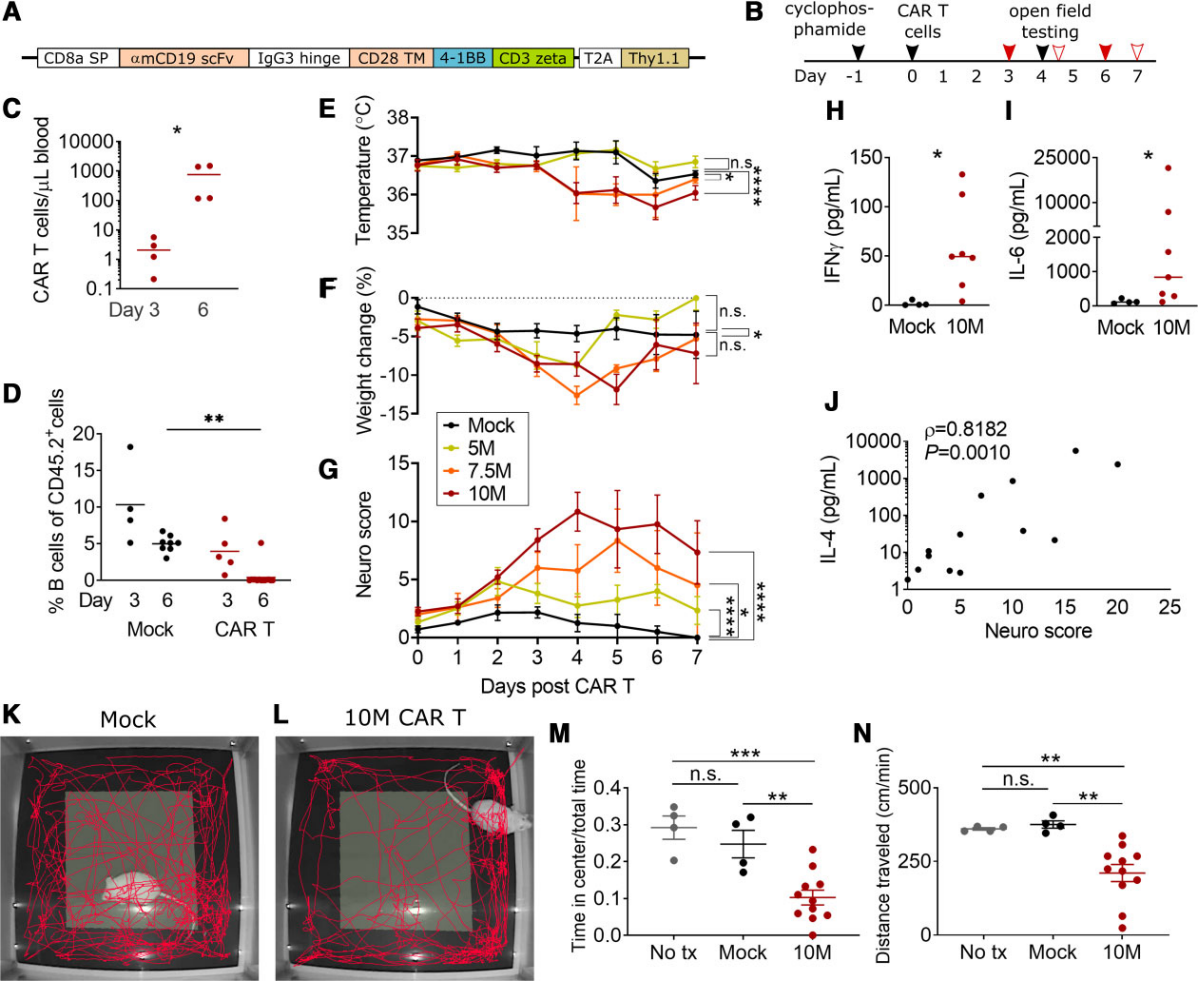
**Neurotoxicity and CRS after CAR T-cell treatment.** (**A**) Structure of the murine CD19-directed CAR. SP, signal peptide; TM, transmembrane domain. (**B**) Experimental scheme. The solid red arrows show the timing of blood draws for flow cytometry; outlined red arrows show blood draws for adhesion molecule and cytokine analysis. (**C**) CAR T cells (CD3^+^ Thy1.1^+^) in blood by flow cytometry. *X*-axis denotes day after CAR T-cell injection. Mice received either 5, 7.5 or 10 × 10^6^ cells/mouse. (**D**) CD19^+^ B cells as a fraction of total CD45^+^ splenocytes. Depletion by CAR T cells is completed by Day 6. (**E**) Ear temperature, (**F**) body weight and (**G**) neurologic exam scores in the first 7 days after CAR T-cell infusion, *P*-values were calculated by linear mixed models (mixed-effects analysis). (**H**) Serum levels of interferon-γ on Day 4 after treatment with 10 million CAR T cells (10M) or control T cells (Mock). (**I**) Serum levels of IL-6. (**J**) Correlation of IL-4 serum levels and same-day neuro score, Spearman’s *r*. Representative examples of 5 min tracks in the open field for a CAR T-cell-treated (**K**) and mock T-cell-treated (**L**) mouse. (**M, N**) Open-field test. ‘No tx’ are naïve control mice who received no cyclophosphamide or CAR T cells. *Y*-axis denotes the fraction of time spent in the centre of the arena (**M**) and locomotion speed (**N**) during the 5 min test. (**C**, **D**, **M**, **N**) Lines show the mean, statistics by one-way ANOVA with the Holm–Sidak post-test. (**H**, **I**) Lines show the median, Mann–Whitney test. All panels: *P < 0.05, **P < 0.01, ***P < 0.001, ****P < 0.0001, all other comparisons are *P* ≥ 0.05. Each data point denotes one animal, except **E–G** where each data point shows the mean of five or more animals per group, with some time points having missing values from some animals. All data pooled from three or more independent experiments.

To explore the mechanism of neurotoxicity during CAR T-cell treatment, we used a syngeneic mouse model of CD19-CAR T-cell treatment (Fig. 1A and B). To avoid confounding effects from tumour growth or lysis, we conducted all experiments in non-tumour-bearing wild-type BALB/c mice, where CD19-CAR T cells target CD19 on normal B cells.^45^ Control mice received syngeneic T cells which underwent mock transduction without viral vector (‘mock T cells’). All mice received cyclophosphamide for lymphodepletion 1 day prior to treatment. CD19-CAR T cells expanded well despite the absence of a tumour target (Fig. 1C) and remained detectable for at least 56 days (Supplementary Fig. S1A). CAR T cells eradicated B cells as expected (Fig. 1D) and B-cell aplasia also persisted for at least 56 days (Supplementary Fig. S1B). Concurrently with the rapid expansion of the CAR T cells, mice developed dose-dependent hypothermia (Fig. 1E) and weight loss (Fig. 1F). We did not observe any fevers, but some animals with severe toxicity developed hypothermia below 34°C.

To measure behavioural changes consistent with neurotoxicity, we adapted a 20-item test (‘neuro score’) based on a neurophenotyping scoring system that was developed for mutant mouse screening.^38^ Doses of 5 × 10^6^ CAR T cells induced only mild elevations in neuro score, whereas 7.5 × 10^6^ and especially 10 × 10^6^ CAR T cells per mouse induced severe behavioural abnormalities that peaked on Days 4 and 5 after CAR T-cell administration (10 × 10^6^ CAR T cells versus mock, *P *< 0.0001, mixed-effects analysis) (Fig. 1G). Here was no effect of sex or age on peak neuro score within any of the dose groups (Supplementary Fig. S2). The most frequent abnormalities on the neuro score were deficits in postural adjustment, balance and exploration. In addition, mice frequently had piloerection, hunched posture and alterations in breathing patterns (fast breathing in milder toxicity, and deeper, more laboured breaths during severe toxicity). We did not observe seizures or focal motor dysfunction.

Since the clearest phenotype was present in the mice receiving 10 million CAR T cells, we focused on this dose level in subsequent experiments. The behavioural abnormalities were accompanied by elevations in serum cytokines. On Days 4 and 5 after CAR T-cell infusion, serum levels of IFN-γ, IL-2, IL-4, IL-5, IL-6 and IL-10 were significantly elevated in mice receiving 10 × 10^6^ CAR T cells, compared to mice, which received mock transduced CAR T cells (Fig. 1H and I and Table 1). We observed marked heterogeneity of serum cytokine levels between individual CAR T-treated mice, even though all animals had detectable CAR T cells and B-cell suppression. Since neurotoxicity scores also varied between individuals, we hypothesized that differences in systemic proinflammatory cytokine profiles could account for the differences in observed neurological phenotype. Indeed, correlation analysis showed a positive association of neurologic exam scores and serum levels of IL-4 (Fig. 1J), IL-5 (Table 1) and IL-10 (Table 1), while there was no correlation of neuro score with serum levels of CXCL1, IL1β, IL-2, IL-6, IL-12p70/IL-25, IFN-γ or TNF (Table 1). Open-field testing on Day 4 after CAR T-cell infusion showed that mice treated with 10 × 10^6^ CAR T cells had markedly decreased locomotion speed (mean 210 versus 375 cm/min for mock, *P *= 0.0060) and spent a smaller fraction of time in the centre of the field (mean 10.3 versus 24.7% for mock, *P *= 0.0043) (one-way ANOVA with Holm–Sidak post-test to correct for multiple comparisons). Assuming constant locomotion speed, the CAR T-treated mice on average walked only 108 cm in the centre field during the 5 min recording time, compared to 463 cm for the mock treatment group (Fig. 1K–N). This indicates that CAR T-treated mice did not only show a decrease in activity level, but also an increase in avoidant or anxious behaviour.

**Table 1.** Serum cytokine levels in non-tumour-bearing mice after CAR T-cell treatment

	10M CAR T (*N* = 7)	Mock (*N* = 4)		Correlation with severity of neurotoxicity (5–10M CAR T) (*N* = 14)	
	Median (pg/ml)	Median (pg/ml)	*P* (Mann–Whitney)	Spearman *r*	*P* (two-tailed)
CXCL1	731.6	386.4	*0.2309*	0.2369	*0.4328*
IFN-γ	**49.3**	**0.6**	***0.0121***	0.0000	*>0.9999*
IL-1β	18.8	2.396	*0.2303*	−0.08264	*0.7885*
IL-2	**3.0**	**0.8**	***0.0242***	−0.2369	*0.4328*
IL-4	**342.8**	**0**	***0.0061***	**0.8182**	***0.0010***
IL-5	**2723**	**13.6**	***0.0242***	**0.8402**	***0.0006***
IL-6	**836.4**	**112.2**	***0.0121***	0.2893	*0.4328*
IL-10	**239.4**	**5.1**	***0.0121***	**0.7466**	***0.0046***
IL-12p70	0	0	*0.6606*	0.3241	*0.2809*
TNF	132.7	43.5	*0.1091*	0.2672	*0.3745*

Left column shows Days 4 + 5 measurements, and right column shows Days 4–7. Significance levels are shown in italics, and comparisons that are statistically significant (*P*<0.05) are shown in bold.

Taken together, these data show that wild-type non-tumour-bearing mice develop acute CRS and abnormal behaviour after treatment with CD19-directed CAR T cells. The timing of the behavioural changes coincides with the period of CAR T-cell proliferation and cytokine release, similar to what occurs in human patients.

### Cerebral microhaemorrhages and focal endothelial disruption are associated with neurotoxicity

**Figure 2:**
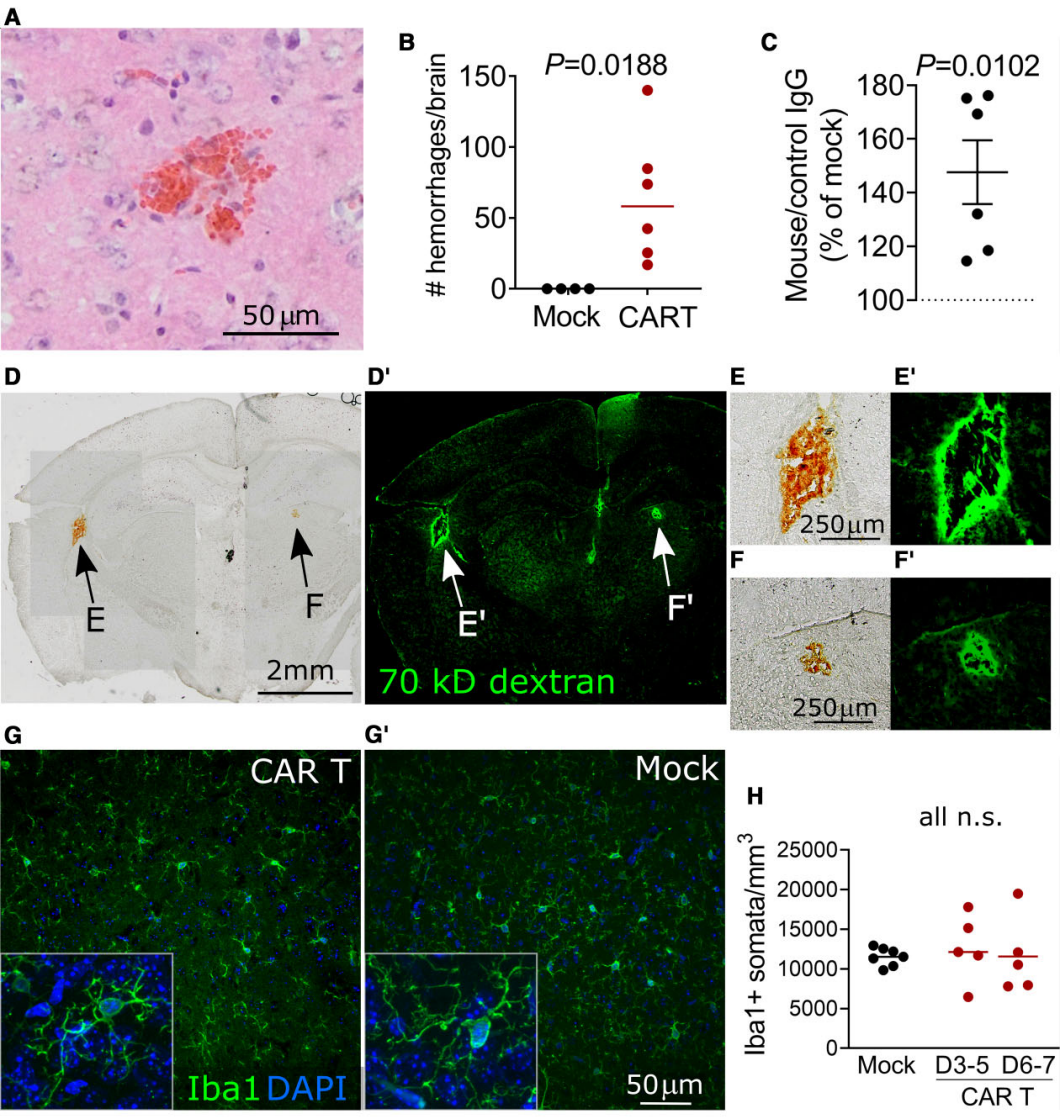
**Cerebral microvascular injury during neurotoxicity**. (**A**) Acute microhaemorrhage in the dorsal cortex. H&E-stained brain section from a mouse that received 10 × 10^6^ CAR T cells 4 days prior. (**B**) Quantification of brain microhaemorrhages in the entire brain. (**C**) Quantification of anti-mouse IgG immunofluorescence normalized to control species IgG immunofluorescence. Each data point shows the median of three brain sections from one mouse, normalized to concurrently treated controls receiving mock transduced T cells. CAR T-cell-treated mice received 5, 7.5 or 10 × 10^6^ CAR T cells 4–6 days prior to sacrifice. (**D**) Bright field image of coronal brain section from a CAR T-treated mouse that received i.v. FITC-dextran 30 min prior to sacrifice. Arrows indicate microhaemorrhages, which are visible without additional tissue staining. (**D′)** Fluorescence imaging of the same section, demonstrating FITC-dextran extravasation surrounding the haemorrhages. (**E′)** Magnification of subcortical haemorrhage in **C**. (**F)** Magnification of basal ganglia haemorrhage. (**E′**, **F′**) The corresponding fluorescence images. (**G**, **G′**) Representative immunofluorescence of microglia in the somatosensory cortex labelled with anti-Iba1 antibody. Insets show individual microglial cells with processes at 40× magnification. In the CAR T-treated mouse (**G**), the microglia maintain a similar finely ramified resting appearance as mock controls (**G′**). (**H**) Quantification of microglial somata counts. All graphs: each data point represents one mouse, pooled data from three experiments and lines show mean. Statistical analyses used: (**B**, **C**) one-sample two-tailed *t*-test. (**H**) One-way ANOVA with Holm–Sidak post-test.

In humans, severe CAR T neurotoxicity can be accompanied by endothelial activation and injury to the cerebral microvasculature.^7^ We examined the brain tissue in our mouse model to determine if similar microvascular changes were occurring. Indeed, we noted cerebral microhaemorrhages in the mice receiving 10 × 10^6^ CAR T cells per mouse (mean 64, range 17–140 haemorrhages per brain, *P *= 0.0188, two-tailed one-sample *t*-test) (Fig. 2A and B). Haemorrhages occurred in all brain regions, including cortex, basal ganglia, hypothalamus, brainstem and cerebellum (Fig. 2). There was no statistically significant correlation between the number of haemorrhages and neuro score on Day 4 (Supplementary Fig. S3). No haemorrhages were seen in mice treated with mock transduced T cells or those receiving the lower doses of 5 × 10^6^ or 7.5 × 10^6^ CAR T cells per mouse.

To determine whether more widespread blood–brain barrier disruption was present even in areas without microhaemorrhages, we used immunohistochemistry to quantify IgG deposition in the cortex as a measure of increased permeability to macromolecules.^46^ CAR T-cell-treated mice had a mean of 47% higher levels of immunofluorescence against mouse IgG compared to mock-treated controls (*P* = 0.0102, one-sample *t*-test) (Fig. 2C and Supplementary Fig. S4). As another measure of blood–brain barrier disruption, we injected 70 kDa fluorescent-conjugated dextran 30 min prior to sacrificing the mice. We observed extensive leakage of dextran around microhaemorrhages, but there was no visible extravasation of dextran in areas without haemorrhage (Fig. 2D–F). Some amount of fluorescently labelled dextran remained visible within the cerebral microvessels even after thorough transcardiac perfusion. This precluded the use of quantification of overall brain dextran fluorescence for measuring blood–brain barrier permeability, since we would not have been able to distinguish between intravascular and extravascular dextran.

Since activation and/or proliferation of brain resident microglia occurs during many neuroinflammatory conditions,^47^ we reasoned that microglial activation may accompany the proinflammatory cytokine environment. Surprisingly, we found normal microglial ramification on histologic examination, as is indicative of a quiescent surveillance state (Fig. 2G). Almost all cortical microglia had processes that were touching or wrapping capillaries, thus preferential activation of perivascular microglia appeared unlikely (Supplementary Fig. S5). There was no difference in the number of Iba^+^ microglia either on Days 3 and 4 (median 13 649 cells/mm^3^) or Days 6 and 7 after CAR T cells (median 11 310 cells/mm^3^) compared to mock-treated control (11 861 cells/mm^3^, *P *= 0.4269) (Fig. 2H).

### Loss of pericyte coverage and increased incidence of string capillaries

**Figure 3:**
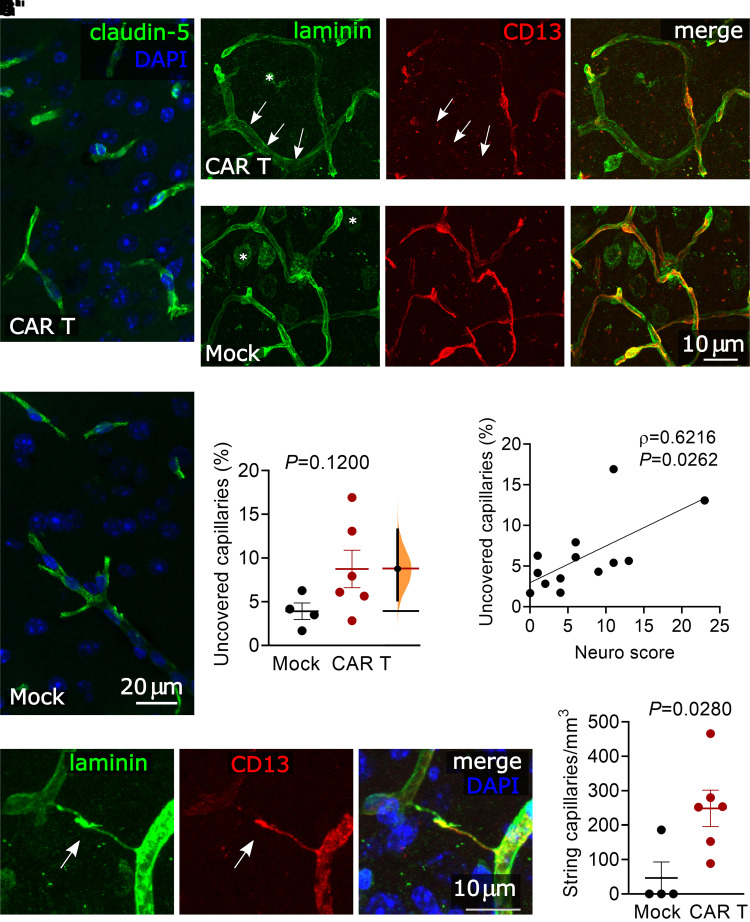
**Pericyte disruption and string capillaries**. All images and analyses are in the somatosensory cortex. (**A**) Representative images of claudin-5 immunofluorescence showing no deficit in endothelial tight junctions in a mouse treated with 10 × 10^6^ CAR T cells 6 days prior, compared to mock T-cell-treated control. (**B**) Representative images of laminin immunofluorescence showing intact capillary endothelial basement membrane in a mouse treated with 10 × 10^6^ CAR T cells 6 days prior (**B**) compared to mock T-cell-treated control CAR T (**B′**). The laminin positive round shapes in the image (examples are labelled by asterisks) are neurons, which were not considered in the analysis. (**C**) CD13 immunolabelling showing pericytes in same areas as **B**/**B′**. Arrows indicate a capillary with loss of pericyte coverage. (**D**) Overlay of laminin and CD13 immunolabelling in same areas as **B**/**B′**. (**E**) Capillary pericyte coverage in mice receiving 10 × 10^6^ CAR T cells compared to mock-treated mice, unpaired two-sided *t*-test. The mean difference between Mock and CAR T is shown in the Gardner–Altman estimation plot. Both groups are plotted on the left axes; the mean difference is plotted on floating axes on the right as a bootstrap sampling distribution. The mean difference is depicted as a dot; the 95% confidence interval is indicated by the ends of the vertical error bar. (**F**) Correlation of pericyte coverage and neurological exam scores for mice receiving 0–10 × 10^6^ CAR T cells. Spearman rank correlation. (**G**) Representative image of a string capillary lacking a lumen (arrow). (**H**) String capillary counts per mm^3^ of cortex in mice receiving 10 × 10^6^ CAR T cells compared to mock-treated mice, unpaired *t*-test. All graphs show pooled data from three experiments, each data point represents one mouse and error bars show mean ± SEM.

Given the observation of microhaemorrhages and increased IgG deposition in the brain, we hypothesized that components of the blood–brain barrier might show morphologic abnormalities even in areas without frank haemorrhage. Claudin-5, a key component of brain endothelial tight junctions,^48^ had unchanged appearance on immunofluorescence on Day 6 after CAR T-cell treatment (Fig. 3A). The capillary endothelial basement membrane also appeared intact, as evidenced by laminin^49^ immunofluorescence which did not show gaps or breaks (Fig. 3B), and covered the same capillary lengths per volume of cortex in CAR T (573 μm/mm^3^) and mock-treated (581 μm/mm^3^) groups (*P *= 0.9329, unpaired *t*-test). However, pericyte coverage of cortical capillaries trended towards a decrease in CAR T-treated animals (Fig. 3C and D). The mean uncovered capillary length was 8.7% of total capillary length in animals receiving 10 × 10^6^ CAR T cells, compared to 3.9% in mock controls, *P *= 0.1200. This phenotype was heterogeneous among animals (Fig. 3E) and the unpaired mean difference between Mock and CAR T was 4.84% (95.0% CI 1.14, 9.41).^50^ To better understand why some CAR T-treated mice had more uncovered capillaries than others, we compared the neurologic symptom score to pericyte coverage and found that mice with more severe neurologic phenotypes also had a greater fraction of uncovered capillaries (Spearman’s *r* = 0.6216, *P *= 0.0262, Fig. 3F). This did not appear to be due to the loss of the pericytes themselves, since the numbers of pericyte cell bodies were similar both as measured by capillary length (CAR T = 14.1 and mock = 9.9 pericytes per millimetre capillary length, *P *= 0.1095, unpaired *t*-test) or by volume of brain tissue (CAR T mean = 7839 and mock mean = 5766 pericytes per mm^3^, *P *= 0.1819, unpaired *t*-test). Since pericyte coverage of microvessels is important for maintaining capillary network structure,^51^ we also searched for evidence of capillary regression. Histologically, involuting microvessel segments can have the appearance of string capillaries, which consist of a basement membrane but lack a patent lumen.^52^ Indeed, string capillaries were significantly more common in CAR T-treated animals (mean 249 string capillaries/mm^3^) compared to mock (46 string capillaries/mm^3^) (*P *= 0.0280, unpaired *t*-test) (Fig. 3G and H).

### *In vivo* imaging shows brain capillary plugging during neurotoxicity

**Figure 4:**
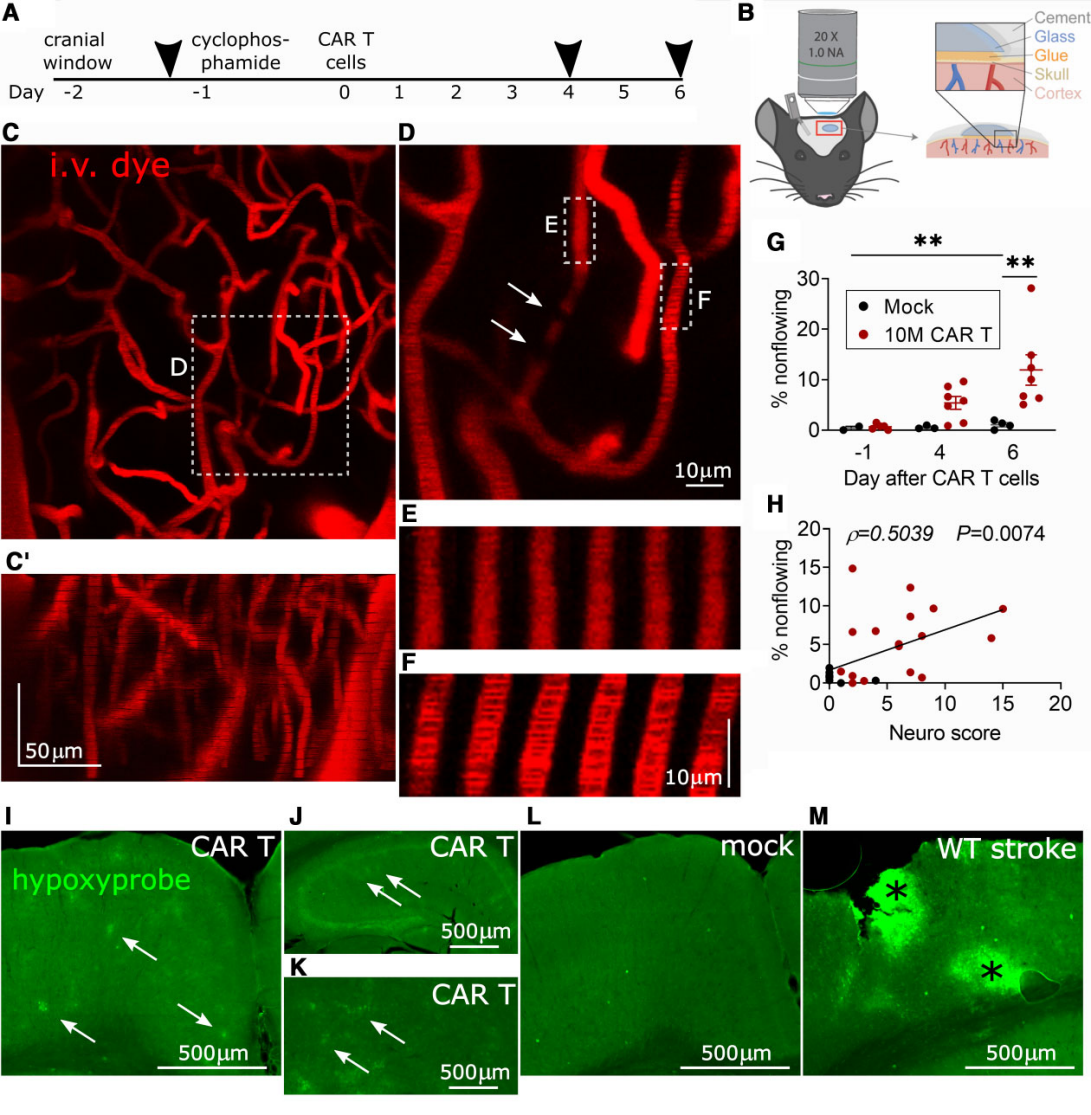
**Cortical capillary plugging visualized by *in vivo* two-photon imaging**. (**A)** Timeline of experiments, arrowheads denote imaging sessions. (**B**) Schematic of thinned-skull *in vivo* imaging preparation. Timeline of experiments **C**, representative image of cortical capillary bed, *z*-projection viewed from top down and **C′** from the side, with the top of the image oriented towards the pial surface of the cortex. (**D**) Close-up of a non-flowing capillary with plugs (arrows) that are seen as filling defects. A section of a non-flowing (**E**) and a flowing (**F**) capillary is shown as a montage of six successive scans. The images are separated by 1.14 s in time and 1 μm in the *z*-axis. In the flowing capillary in **F**, moving shadows of red blood cells can be seen, which are absent in **E**. (**G**) Percentage of non-flowing cortical capillaries during each of the imaging time points. **P < 0.01, one-way ANOVA with the Holm–Sidak post-test, bars show mean ± SEM. Each data point represents one animal, data from three separate experiments. (**H**) Correlation of behavioural neuro score on day of imaging and percentage of non-flowing capillaries, Spearman correlation. Combined data from Days 4 and 6 after treatment. (**I–M**) Hypoxyprobe immunofluorescence in coronal brain sections, pial surface is at the top: (**I**) patches of hypoxic cortex in a CAR T-cell-treated mouse (arrows). Patchy hypoxia is also visible in hippocampus (**J**, note different scale bar) and thalamus (**K**). In mock-treated control cortex, there is no such patchy hypoxyprobe labelling (**L**). (**M**) Positive control. A small stroke was made by laser coagulation of a cortical venule, resulting in two areas of hypoxia (asterisks).

We next sought to determine whether the abnormalities seen on histology correspond to functional changes in the perfusion of the cerebral microvasculature. To measure cerebral capillary blood flow, we performed *in vivo* two-photon imaging during the period of the most severe behavioural neurotoxicity (Days 4 and 6) (Fig. 4A). We used a thinned-skull cranial window preparation to minimize surgically induced inflammation of the underlying meninges and brain (Fig. 4B). We acquired ∼150 μm deep *z*-stacks of the somatosensory cortex to analyse the anatomy and flow patterns of the microvasculature. Each *z*-stack was collected prior to CAR T-cell treatment and on Days 4 and 6 post-treatment. At none of the time points did we observe any leakage of intravascular dextrans into the brain parenchyma. Strikingly, 5.4% of cortical capillaries were non-flowing on Day 4, which appeared to be due to cells plugging the capillaries (Fig. 4C–F). We only considered plugs lasting >5 s, since momentary stalls can be caused by large leucocytes even in normal conditions. On Day 6, the fraction of plugged capillaries increased to 11.9%, compared to 1.1% in controls (*P *= 0.0072) (Fig. 4G). This level of capillary plugging is very severe and likely to cause behavioural abnormalities, as only 2% capillary plugging was sufficient to cause cognitive abnormalities in a mouse model of Alzheimer’s disease.^53^ The abnormal capillary blood flow did not appear to be related to poor systemic perfusion or oxygenation: mean peripheral oxygen saturation measured by paw infrared sensor during imaging was 86% (range 75–91%) versus 85% in mock controls (range 79–95%), and the mean heart rate was 479 beats per minute (range 416–545) versus 475 in mock controls (range 312–542). ECG showed preserved cardiac output (mean 13.3 ml/min in CAR T, 14.5 ml/min in mock on Day 4, *P *= 0.7506; 18.2 ml/min CAR T, 16.7 ml/min mock on Day 6, *P *= 0.8583; unpaired *t*-test; *N* = 5 CAR T, *N* = 2 mock).

Consistent with capillary plugging being a contributor to behavioural abnormalities, neuro scores were higher in mice with more severe capillary plugging (*P *= 0.0074, Spearman’s correlation) (Fig. 4H). Further supporting the pathologic effect of capillary plugging, hypoxyprobe immunolabelling was abnormal in CAR T-cell-treated mice. Patches of hypoxic cells were apparent throughout the brain, including the cortex, but also in deeper regions such as the thalamus and hippocampus (Fig. 4I–K). The loss of capillary pericyte coverage was more severe in cortical areas that labelled strongly with hypoxyprobe (Supplementary Fig. S6). No hypoxia labelling was seen in control mice (Fig. 4L). The specificity of hypoxyprobe labelling was confirmed in a positive stroke control (Fig. 4M).

### Capillary plugs are leucocytes

**Figure 5:**
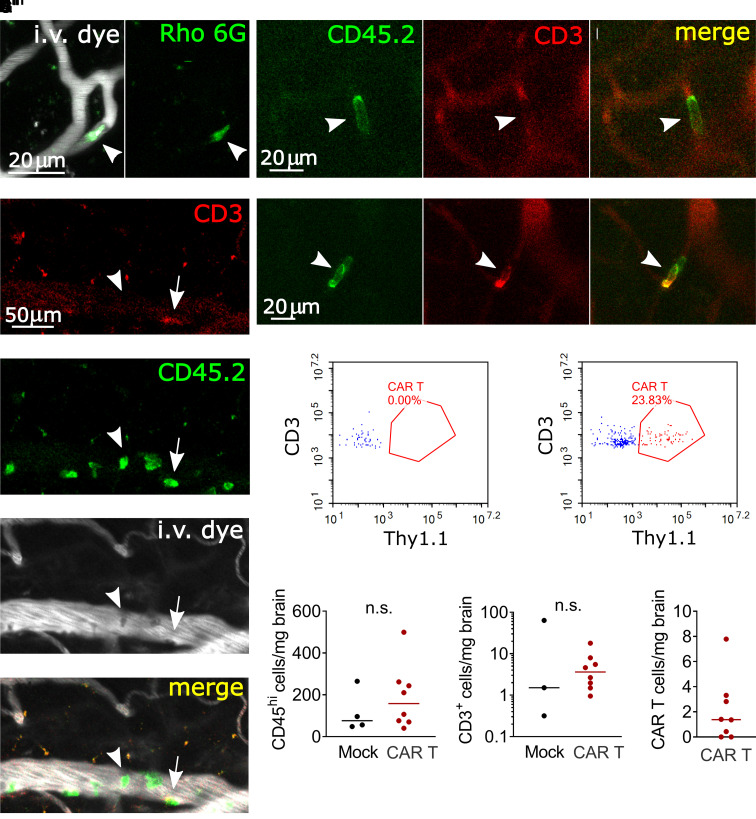
**Leucocyte plugging of cortical capillaries**. (**A**) Representative image of a Rhodamine 6G positive capillary plug. (**B**) Pial venule with multiple leucocytes adhering to the endothelium. Most were CD45.2^+^, CD3^−^ (arrowhead), with scattered T cells (CD45.2^+^, CD3^+^, arrow) **C**, CD45.2^+^, CD3^−^ leucocyte (arrowhead) that remained stationary in a capillary over a 3 min time scan. (**D**) CD45.2^+^, CD3^+^ T-cell plugging a capillary. In **C** and **D**, vessels are outlined in the red channel by circulating unbound anti-CD3-Alexa 594 antibody. (**E, F**) Representative flow cytometry plots of brain-infiltrating untransduced T cells (blue) and CAR T cells (red) in mice treated with mock (**E**) and CAR (**F**) T cells. Thy1.1 is the transduction marker coexpressed with the CAR. (**G–I**) Flow cytometry of brain-infiltrating leucocytes on Days 3–6 after treatment. Each data point represents one mouse, mice received 5–10 × 10^6^ CD19-CAR T cells or 10 × 10^6^ mock transduced T cells, pooled data from three replicate experiments. Lines show the median. Mann–Whitney test.

To identify the cause of the capillary plugging, we first used i.v. injections of Rhodamine 6G,^54^ which labels leucocytes and platelets. Capillary plugs were consistently Rhodamine 6G positive (Fig. 5A). To further confirm the identity of the plugs, we injected fluorescently conjugated antibodies during imaging (anti-CD45.2 as a pan-leucocyte label and anti-CD3 to identify T cells). In CAR T-treated mice, but not in mock controls, we observed large numbers of crawling or firmly adherent CD45.2+ leucocytes in pial venules, a subset of which was also positive for CD3 (Fig. 5B). All capillary plugs labelled with anti-CD45.2 antibody and many were double positive for CD45.2 and CD3 (Fig. 5C and D). This suggests that leucocyte–endothelial adhesion is increased globally affecting larger vessels as well as capillaries, and that not all adherent cells are CAR T cells. Given the observation of increased crawling, adhesion and capillary stalling by T cells and other leucocytes, we next asked whether CAR T-cell treatment leads to increased migration of peripheral leucocytes into the brain parenchyma. Flow cytometry of enriched brain leucocytes showed no significant increase of immune cell infiltration in CAR T-cell-treated mice (Fig. 5E–G) (median 158 CD45^hi^ cells/mg brain tissue) compared to mock control (median 76 CD45^hi^ cells/mg) (*P *= 0.5697, Mann–Whitney test). The number of infiltrating T cells was also not elevated, with 3.6 CD3+ cells per milligram brain tissue compared to 1.5 CD3+ cells in controls (*P *= 0.7758, Mann–Whitney test) (Fig. 5H). CAR T-treated mice had a median of just 1.4 CAR T cells/mg brain (Fig. 5I). Taken together, these data support the theory that intravascular obstruction by leucocytes is a key event in the pathogenesis of CAR T neurotoxicity.

**Figure 6:**
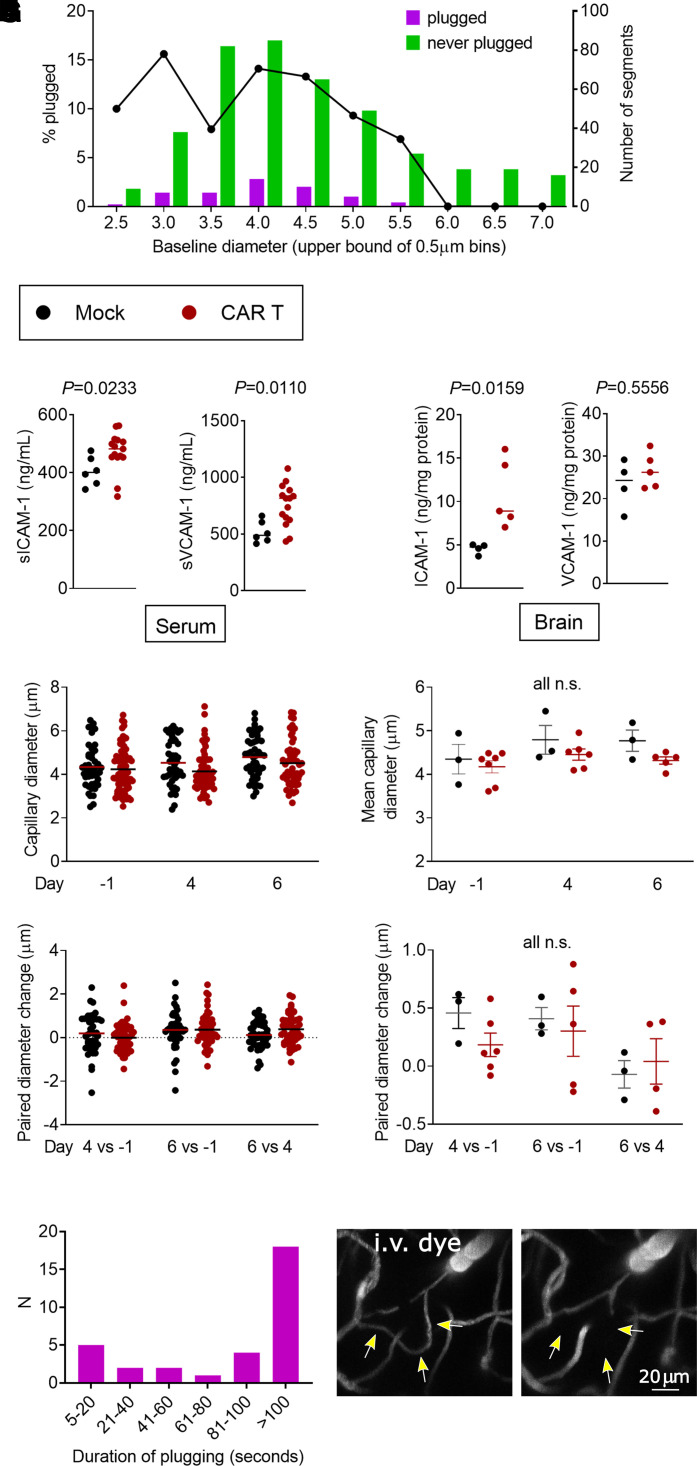
**Adhesion molecule expression and kinetics of capillary plugging**. (**A)** Baseline diameters (Day 1) and likelihood of plugging on Days 4 and/or 6. Left *y*-axis and line plot show the percentage of capillaries that had any plugging. The right *y*-axis and bar graphs show the raw counts of capillary segments in each size bin. Each bar represents a single measurement from pooled data from six mice, each treated with 10 million CAR T cells and Days 4 and 6 time points. (**B–G**) Black dots, mock; red dots, CAR T-cell-treated mice. (**B**) Serum levels of soluble VCAM-1 and VCAM-1. (**C**) Protein content of ICAM-1 and VCAM-1 in brain lysates. For **B** and **C**, each data point represents one mouse, the lines show the median, *P*-values per Mann–Whitney test. (**D**) Representative example of capillary diameters in one CAR T (red) and one mock (black)-treated mouse. Each data point represents one capillary segment, all segments <7 μm baseline diameter in 3 *z*-stacks were included. Line denotes the mean diameter. (**E**) Summary of mean capillary diameters from three separate experiments, each data point represents one mouse. Lines show mean and SEM, one-way ANOVA. (**F**) Representative example of diameter changes in individual capillary segments in the same animals as in **D**, each data point is one capillary segment and line denotes the mean. (**G**) Summary plot of diameter changes over time, each data point shows the mean from one mouse and lines show mean and SEM, one-way ANOVA. (**H**) Duration of plugging. Bars show the number of capillary plugs that persisted for the amount of time indicated on the *x*-axis. For plugs lasting <100 s, both the beginning and the end of the stalling event were observed. Each bar represents a single measurement from pooled data from five mice at Days 4 and 6 time points. (**I**, **J**) Rare loss of a capillary segment. Arrows show a flowing segment on Day 1 (**I**), which no longer fills with dye on Day 6 (**J**).

We next considered the role of capillary diameter in the aetiology of capillary obstruction by leucocytes. To determine if the cause of plugging might be the inability of activated, enlarged leucocytes to transit smaller capillaries, we measured the baseline (pre-treatment) diameters of the capillaries that later developed plugs. Only capillaries with a baseline diameter of <5.5 μm developed plugs (Fig. 6A). This is consistent with our observation that all plugging objects are leucocytes, which are likely too small to obstruct any of the larger vessels. If simple mechanical obstruction was the cause of plugging, then the smaller a capillary, the more likely it should end up being plugged by a circulating leucocyte. But the likelihood of plugging remained similar in the size range from 2.5 to 4.5 μm (Fig. 6A) and leucocytes were able to deform to fit through small capillaries (Fig. 5C and D). To lend further support to a mechanism based on increased cell adhesion as opposed to mechanical plugging, we found that serum levels of soluble ICAM-1^55^ and VCAM-1^56^ are elevated in CAR T-cell-treated mice (median 482 versus 401 ng/ml, *P *= 0.0233 and 815 versus 589 ng/ml, *P *= 0.0110, respectively; Mann–Whitney test) (Fig. 6B). This is consistent with the increased expression of leucocyte adhesion molecules by the endothelium. In the brain parenchyma, we found higher amounts of ICAM-1 protein in CAR T-treated mice (8.90 ng/mg protein versus 4.76 ng/mg protein in mock, *P *= 0.0159). There was no difference in VCAM-1 protein amounts in the brain (26.2 ng/mg protein in CAR T-treated versus 24.3 ng/mg protein in mock, *P *= 0.5556) (Fig. 6C). We considered the possibility that systemic cytokine release could globally decrease capillary diameters, thereby increasing the fraction of capillaries smaller than 5.5 μm and increasing the likelihood of plugging. However, there was no statistically significant difference in capillary diameters on Days 1, 4 and 6 (Fig. 6D and E), diameter change between imaging days (Fig. 6F and G) or variance of diameter changes (Supplementary Fig. S7) between CAR T and mock-treated mice. Taken together, these data best support the conclusion that increased leucocyte adhesion to the endothelium mediates capillary plugging.

To better understand the kinetics of leucocyte stalling, we obtained time-series images of randomly selected capillary plugs. Of 32 stalls observed for at least 100 s, 44% resolved within the 100 s observation time frame, which would be most consistent with leucocyte rolling or crawling (Fig. 6H). However, since more than half the plugging cells remained stationary for over 100 s, we reasoned that such prolonged interruption of blood flow might cause regression of the vessel. To test this, we first identified 31 capillary segments that were plugged on Day 4 and were well visualized again on Day 6. Of the initially plugged segments, 74% were recanalized and flowing on Day 6, 26% remained plugged and none had regressed. Considering the possibility that we did not see regression because many plugging events occurred outside of the imaging time frame, we next assessed the patency of all capillary segments between baseline and Day 6 after CAR T treatment. If our histologic finding of ∼250 string capillaries per mm^3^ (Fig. 3G and H) were to be directly reflective of capillary segments that lost patency after CAR T-cell treatment, we would expect to find capillary regression at the same frequency during *in vivo* imaging (0.75 regressing capillary segments per 0.003 mm^3^  *z*-stack). However, when considering all capillary segments that we were able to follow from baseline imaging to Day 6, whether later plugged or not, we were able to identify loss of any capillaries in only two of seven CAR T-cell-treated mice (3/272 segments, 1.1%, in one mouse; 1/243 segments, 0.4%, in the other). We considered vessels as regressed if the entire segment had dropped out instead of having a cell-shaped filling defect (Fig. 6I and J). We found no loss of capillary segments in any of the mock control mice.

These findings show that despite the prolonged obstruction of capillaries, regression occurs only in a small fraction of segments during the acute phase of neurotoxicity. The discrepancy between the frequencies of string capillaries on histology and capillary segment loss on *in vivo* imaging may be due to undersampling, since not all capillary segments could be visualized at all three *in vivo* imaging time points. However, they may also represent mechanistically distinct phenomena.

## Discussion

We have developed a mouse model which recapitulates key aspects of clinical CAR T-cell neurotoxicity—behavioural changes, association with CRS and injury to cerebral microvessels. Using *in vivo* two-photon imaging, we discovered that plugging of cortical capillaries by leucocytes is a putative mechanism underlying neurotoxicity.

In our model, neurotoxicity begins during the first week after CAR T-cell infusion, concurrently with CRS or very soon thereafter. Similarly, patients receiving CD19-directed CAR T cells typically develop neurotoxicity within days of the onset of CRS, often before CAR T cells are detectable in large numbers in the peripheral blood.^10–12^ This may be explained by the fact that cytokine elevations are highest during the initial expansion of CAR T cells *in vivo*,^10,20^ or possibly by a model where the presence of very few CAR T cells is sufficient to trigger a monophasic neurologic injury. This close temporal association of neurotoxicity differs from a prior model of CD19-CAR toxicity, where human haematopoietic stem cells and human CAR T cells were engrafted into immunodeficient mice transgenic for human haematopoietic growth factors (NSG-SGM3).^22^ Here, neurologic abnormalities did not develop until 4 weeks after CAR T-cell infusion, when CRS had already resolved. In addition, histopathologic changes in this model consisted of meningeal infiltration of human macrophages, but no cerebrovascular changes were shown. These differences may be due to the fact that our syngeneic model allows murine immune mediators to interact with the murine neurovascular unit, setting into motion a signalling cascade that results in increased immune cell adhesion to the endothelium. Building on prior syngeneic models,^30^ we were able to induce toxicity by increasing the doses of CAR T cells and lymphodepleting chemotherapy. The doses needed to induce toxicity in mice are higher per kilogram body weight than the typical maximum tolerated doses in human patients (for example, doses above 1–2 × 10^6^ CAR T cells/kg typically induce excessive CRS and/or neurotoxicity in acute lymphoblastic leukaemia).^57,58^ It remains unknown why toxicity is dose dependent, since lower doses of CAR T cells can successfully engraft, expand and eradicate tumours.^24^ Higher doses may allow more rapid CAR T-cell proliferation and higher cytokine release, and indeed we noted a correlation between cytokine levels and neurologic toxicity in our model. In addition, there was batch-to-batch difference in the toxicity of CAR T cells, which caused additional variability in clinical toxicity and cytokine levels. This occurred despite accounting for transduction efficiency when calculating CAR T-cell doses and using mice from the same supplier and genetic background. Causes for this could include immunologic differences caused by variability in donor and recipient microbiomes or differences in transgene integration events and CAR expression levels.

We found that over 10% of cortical capillaries were obstructed by adherent leucocytes during acute neurotoxicity. Blood cell velocity and flux might be higher than baseline in neighbouring patent capillaries, as they would take on additional blood cells from otherwise plugged routes in the capillary network. However, on the network level, we would hypothesize that plugging of 10% of the capillaries would create greater flow resistance and reduced tissue oxygenation. Capillary plugging has previously been described as a putative mechanism of neurologic injury in a mouse model of Alzheimer’s disease, where obstruction of only 1.8% of capillaries by stalled neutrophils was correlated with neurologic dysfunction.^53^ The authors used mathematical modelling to show that the occlusion of 2% of capillaries was sufficient to reduce overall cerebral blood flow. While the depth of two-photon imaging only allows us to visualize the microvasculature of the cortex, we found that patchy hypoxia by hypoxyprobe labelling extended into deeper brain regions, including the hippocampus and thalamus. This suggests that capillary stalling is not restricted to the cortex. Regional differences in blood flow may account for the fact that some mice had high plugging but a relatively benign neurologic exam and vice versa.

We propose that capillary plugging is the result of increased expression of adhesion molecules on the endothelium and/or leucocytes in response to proinflammatory cytokine signalling. If the plugging was a purely mechanical process caused by increased size or stiffness of circulating leucocytes, we would have expected an inverse relationship of capillary size and likelihood of plugging, which we did not find. We demonstrated increased serum levels of soluble VCAM-1 and ICAM-1, which have been shown to mediate leucocyte infiltration in the brain during inflammatory conditions.^59–61^ VCAM-1 and ICAM-1 expression is in turn induced by secreted proinflammatory cytokines.^62,63^ Since the expression of adhesion molecules varies between vascular zones,^64,65^ an important next question would be to determine whether there is a predilection for plugging in specific branching orders of the cerebral microvasculature. Further studies should also examine the roles of different leucocyte adhesion molecular mechanisms, since we found that protein levels in brain tissue were elevated for ICAM-1 but not for VCAM-1.

As additional evidence for increased adhesiveness between leucocytes and endothelium, we noted high levels of leucocyte rolling and adhesion in larger venules that were too large to be obstructed. This is consistent with previous *in vitro* studies, which have shown increased endothelial adhesion of T cells that were treated with the bispecific CD3–CD19 T-cell engager blinatumomab.^66^ In patients, blinatumomab can cause a similar neurotoxicity syndrome compared to the one seen with CAR T cells, and our data support a possible unifying mechanism of toxicity between different cell-based immunotherapy modalities. Thus, targeting adhesion molecules may provide a therapeutic modality for preventing or ameliorating neurotoxicity.^67^ Additionally, if one specific cell type is primarily responsible for the plugging, selective ablation may be helpful, while taking care to preserve the therapeutic potential of CAR T cells. For example, in mouse models of ischaemic stroke, over 50% of capillaries in the core and penumbra of the stroke were plugged by neutrophils, erythrocytes or platelet-rich clots, and the plugging could be ameliorated by antibody-mediated ablation of neutrophils.^68,69^ Further identification of the stalling cells in our model by *in vivo* two-photon microscopy will require strong, cell-type-specific fluorescent labelling. Unfortunately, the distinction between CAR-expressing and CAR-negative T cells was not possible with the antibody against our Thy1.1 transduction marker as it did not yield a sufficiently bright signal for *in vivo* imaging. Stable fluorescent labelling can also be accomplished by the adoptive transfer of genetically modified cells or the use of transgenic mice as T-cell donors. The use of transgenic or knockout mice in syngeneic CAR T models is complex, since extensive backcrossing is required to achieve a uniform genetic background to avoid graft-versus-host disease.^27,70^

In addition to *in vivo* capillary plugging, we found histologic evidence of injury to the neurovascular unit, including loss of coverage by capillary pericytes, increased capillary regression and microhaemorrhages. More work remains to be done to understand how these findings are related and whether alterations of paracellular or transcellular transport, tight junction integrity or basement membrane structure may contribute to changed permeability of the blood–brain barrier during CAR T neurotoxicity. Based on the frequency of string capillaries observed on histology, we would have expected to find frequent examples of capillary regression on *in vivo* imaging between baseline and Day 6 post-treatment, but these were actually quite rare. This suggests that either *in vivo* imaging underestimated the number of regressed vessels or that string capillaries represent a different biological entity. Further, pericyte coverage was not examined *in vivo* and the relationship between pericyte loss and capillary diameter could not be established.

A role for direct on-target toxicity by CD19-CAR T cells was suggested by a recent study which noted the expression of CD19 in foetal human brain vascular mural cells by single-cell RNAseq.^71^ This mechanism is plausible, as the destruction of pericytes and other vascular mural cells by CAR T cells could lead to compromise of the blood–brain barrier or changes in blood flow.^72,73^Although we found reduced coverage of capillaries by pericyte processes, the number of pericytes was unchanged in CAR T-treated mice. Additionally, single-cell gene expression analysis of the mouse neurovascular unit shows low to undetectable CD19 expression in pericytes or vascular smooth muscle cells.^64,74^ We did observe gaps in the coverage of pericyte processes in mice with severe behavioural neurotoxicity, the mechanism and consequences of which remain to be explored. Other mechanisms of injury may include cytokine-mediated alterations of neurovascular unit integrity,^75^ or direct insults to the endothelium by stalled immune cells. This may be similar to what occurs during microvascular leukostasis caused by circulating leukaemic blasts, which can be associated with microhaemorrhages even in the absence of overt coagulopathy, indicating that the focal stalling causes loss of endothelial integrity.^76^ Since all the capillary obstructions were CD45.2+ leucocytes and not platelet-rich thrombi, our model does not support a role for thrombotic microangiopathy in the development of CAR T neurotoxicity.^77^

In summary, we have found evidence of neurovascular unit injury and leucocyte plugging of the cerebral microvasculature in a mouse model of CD19-CAR T-cell therapy. This model can serve as a novel platform for dissecting the molecular mechanisms of CAR T-cell toxicity and may be useful in the development of safer cancer immunotherapies.

## Supplementary Material

fcab309_Supplementary_Data

## Abbreviations

CAR =: chimeric antigen receptor
CRS =: cytokine release syndrome
IFN-γ =: interferon gamma
IL =: interleukin
TNF =: tumour necrosis factor
