# Supplementary material for: Brain capillary obstruction during neurotoxicity in a mouse model of anti-CD19 chimeric antigen receptor T-cell therapy
Source: Brain Commun. 2021 Dec 31;4(1):fcab309. doi: 10.1093/braincomms/fcab309 (PMC8833245; doi:10.1093/braincomms/fcab309)
Supplement: fcab309_Supplementary_Data [file fcab309_supplementary_data.docx]

**Supplementary Figures**

**
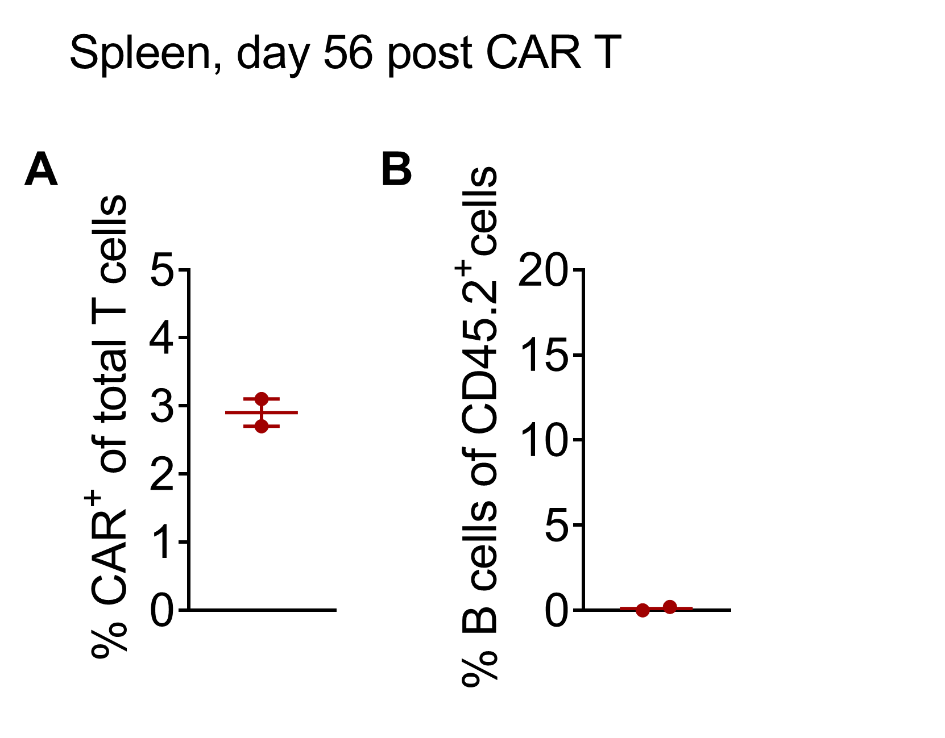
**

**Supplemental Figure S1. CAR T cells and B cell suppression persist for weeks.** Persistence of CAR T cells and B cell suppression on day 56 after infusion of 10 x 10^6^ CAR T cells. Each data point represents one mouse. The y axis in B) is the same as in Figure 1D for ease of comparison.

**
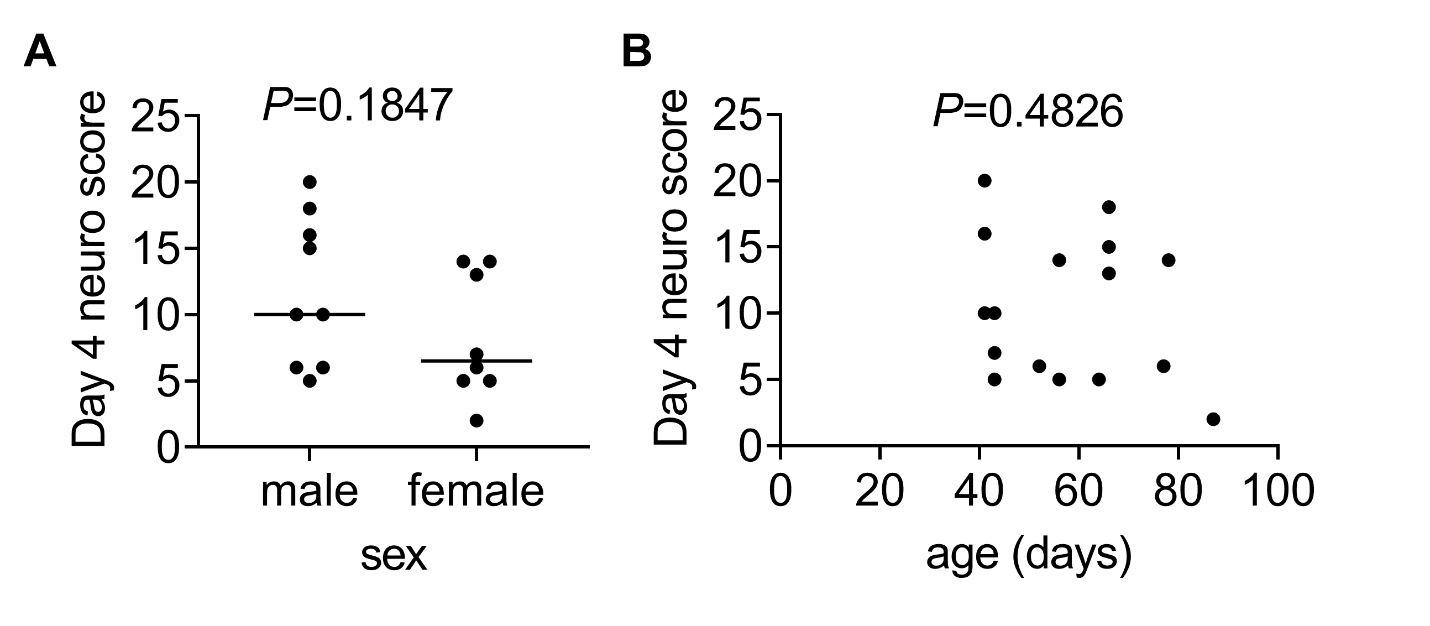
**

**Supplemental Figure S2. Sex and age do not affect neurotoxicity.** A) Male and female mice were randomly chosen to receive CAR T cells or mock transduced T cells. The figure shows CAR T cell recipients only. The day 4 neurophenotype score is shown on the y axis. Unpaired two-tailed t test. B) Age at CAR T cell treatment ranged from 6 to 10 weeks. There was no effect of age on day 4 neurophenotype score by linear regression analysis.


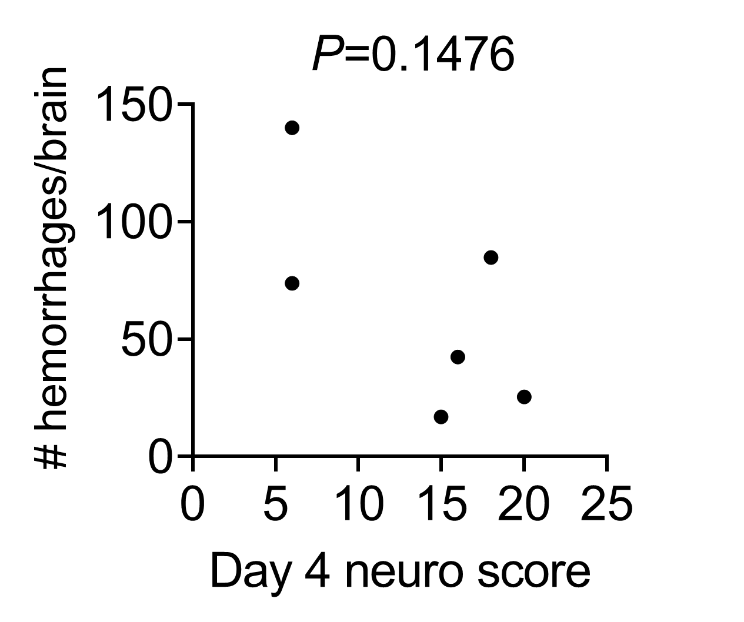


**Supplemental Figure S3. There is no correlation between severity of neurologic deficit and number of brain hemorrhages.** The x-axis shows the neurological exam score on day 4 after CAR T cell infusion, and the y-axisi shows the total number All mice received 10 million CAR T cells per mouse, each data point shows one mouse, Pearson’s correlation.


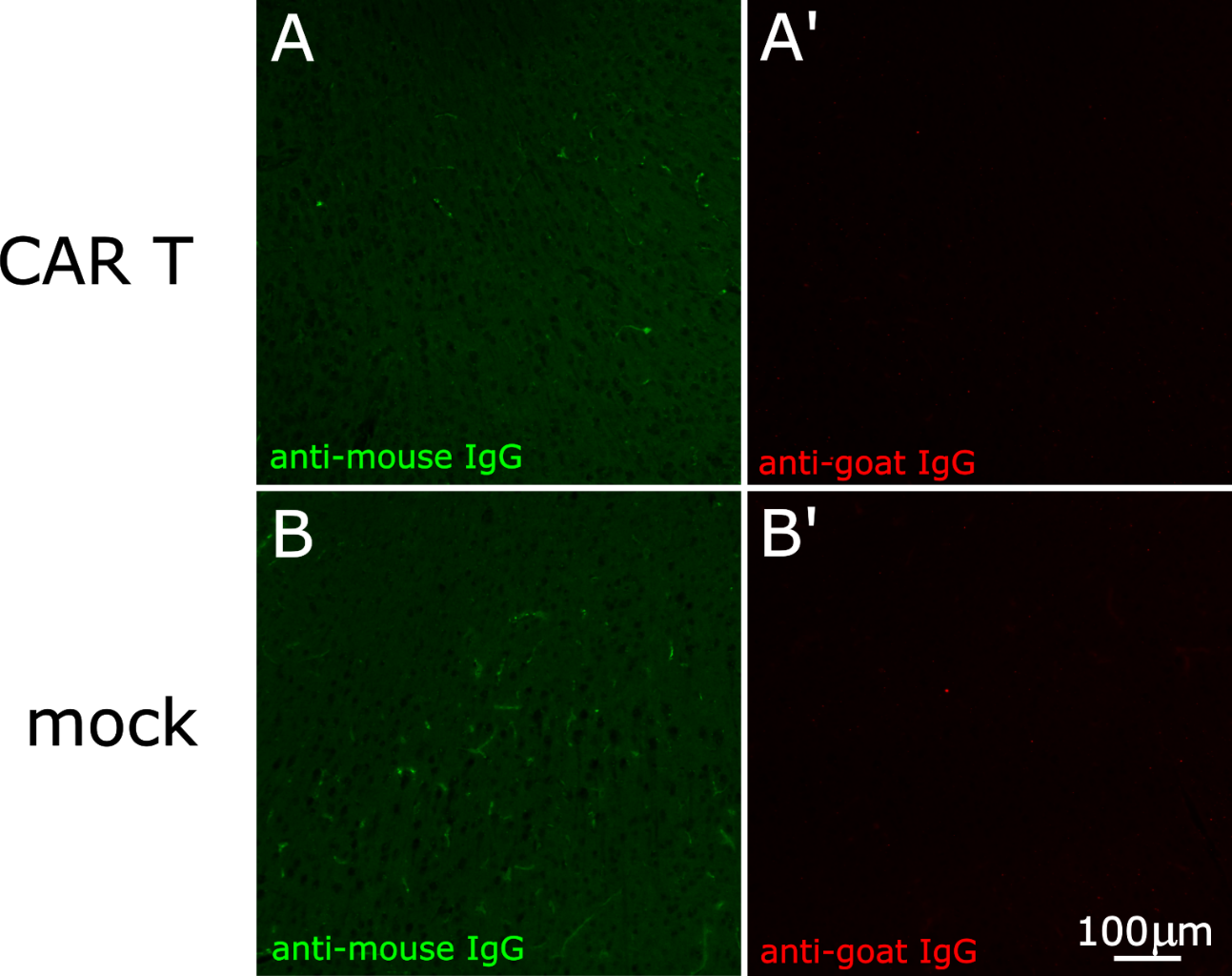


**Supplemental Figure S4. Immunoglobulin deposition in cortex.** Representative images from CAR T (A, A’) and mock (B,B’) T cell treated mice. Brain sections were incubated with fluorescently conjugated anti-mouse or anti-goat IgG antibodies, and mouse/goat fluorescence ratio calculated. In this example, the CAR T mouse/goat fluorescence ratio was 14% higher than in the mock control. The bright green fluorescent spots in A) and B) represent autofluorescent red blood cells.


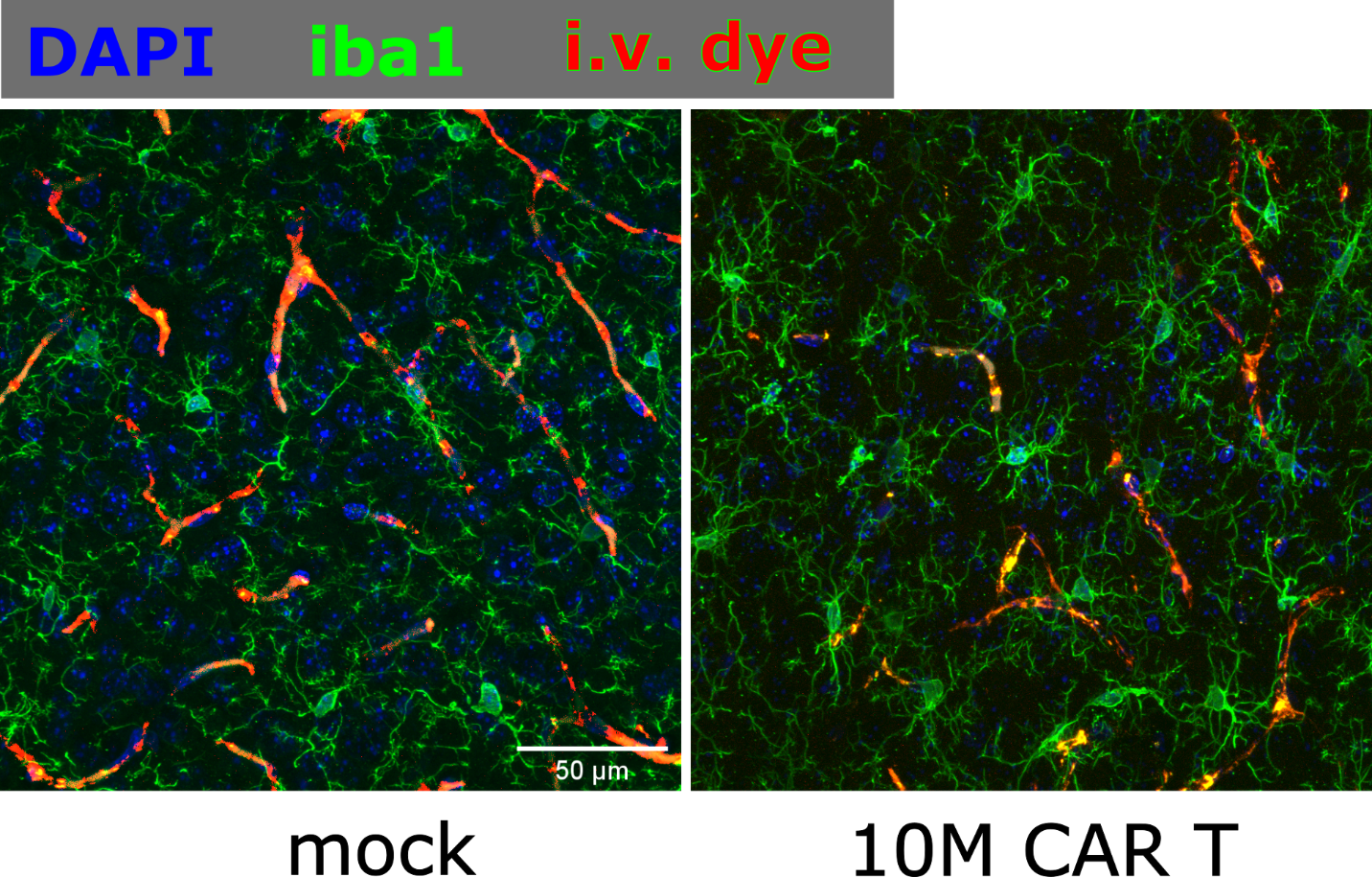


**Supplemental Figure S5. The majority of cortical microglial processes is associated with capillaries**. 50μm brain sections were immunostained with anti-iba1 antibody to label microglia, and capillaries were labeled by intravenous injection of fluorophore-conjugated 70kDa dextrans (mock=tetramethylrhodamine only, CAR T=tetramethylrhodamine and FITC, thus vessels are dual-labeled). In both the mock and the CAR T treated mouse, the majority of microglia have processes that overlap with capillaries in the 2D projection.


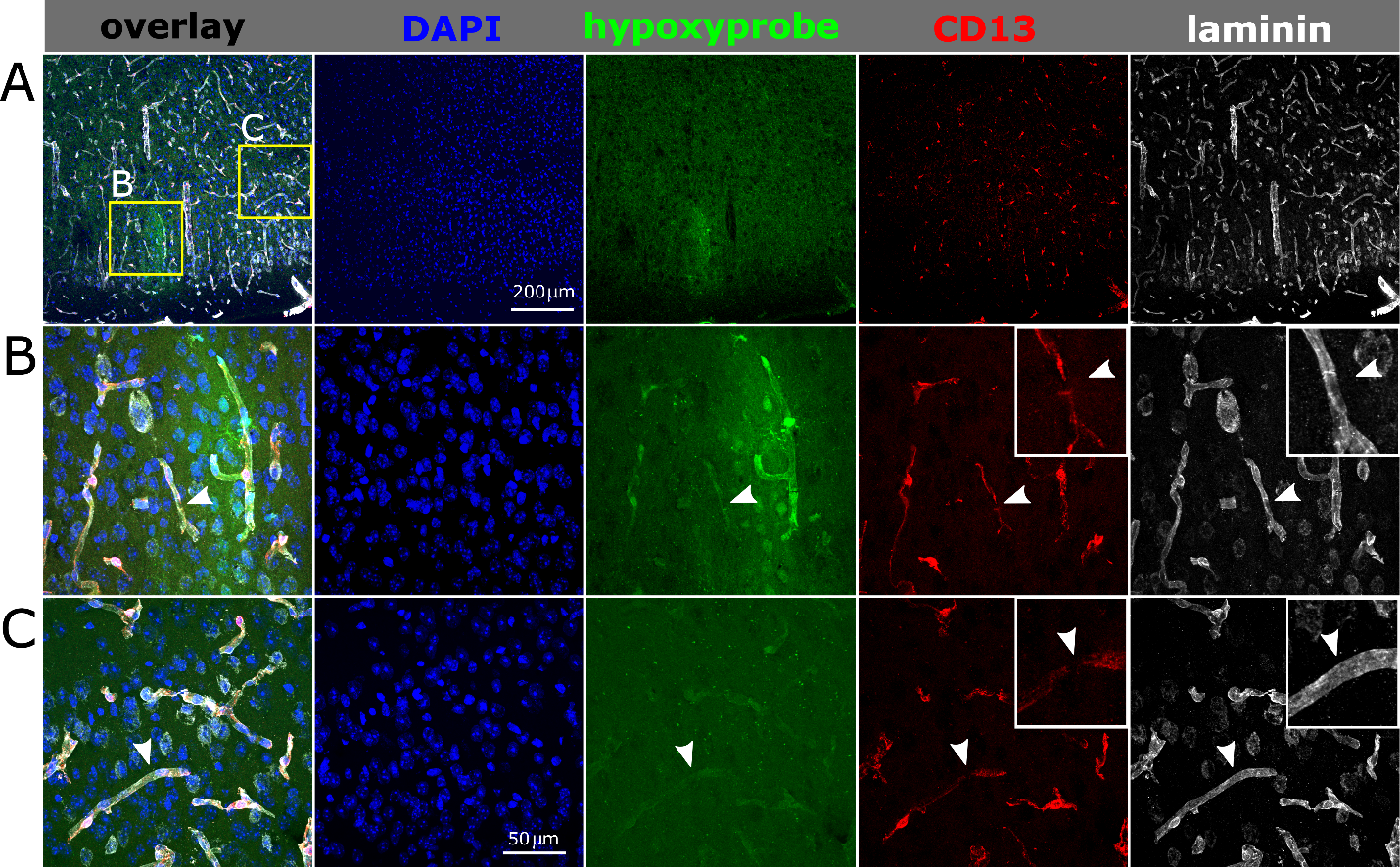


**Supplemental Figure S6. Loss of capillary pericyte coverage is more severe in cortical areas that label strongly with hypoxyprobe.** **A**, overview image taken at 10x magnification. **B**, area with strong hypoxyprobe label, 40x. **C**, area with weaker hypoxyprobe label, 40x. The arrowheads point toward breaks in pericyte coverage, seen in the red channel (CD13). The insets show higher magnification of the same area. In the hypoxyprobe-high regions, 12.2% of laminin+ capillary length was not covered by pericyte processes, in the hypoxyprobe-low areas 7.2% of capillary length was uncovered. Data averaged from 4 or more separate 40x images per condition, N=1 mouse. Analysis was conducted blinded to hypoxyprobe staining.


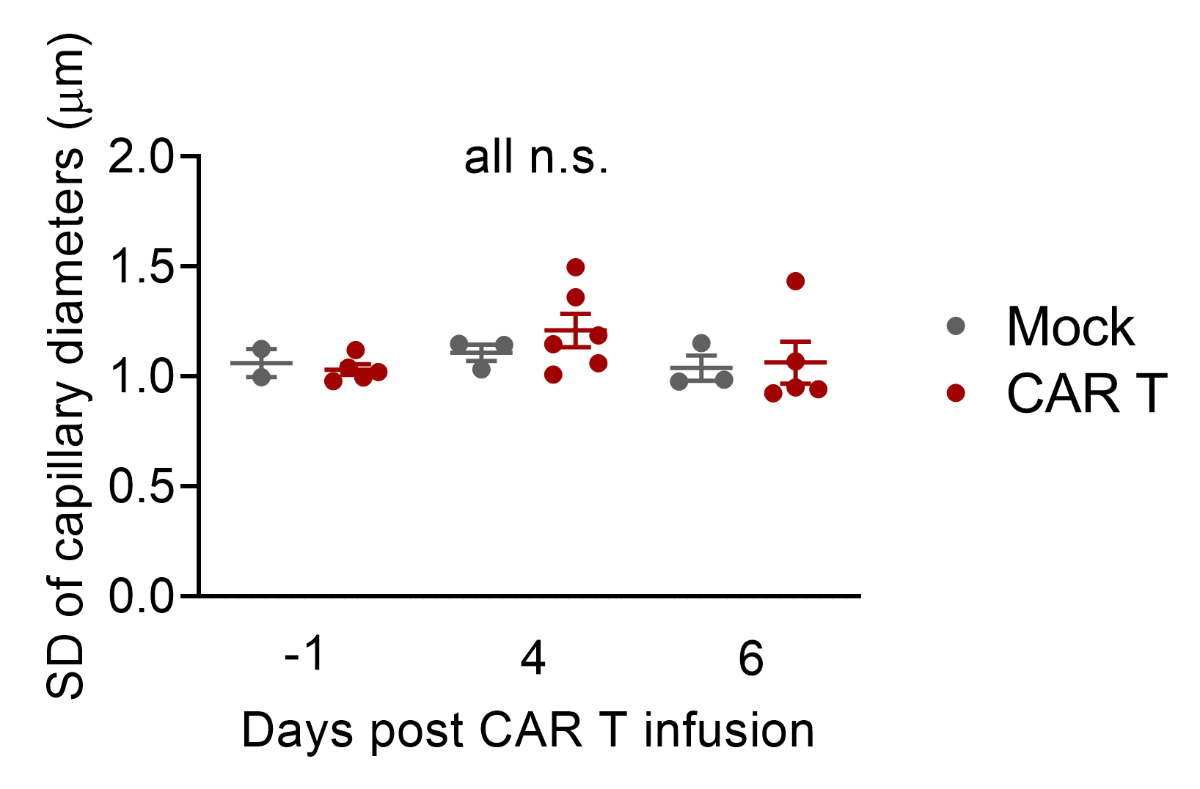


**Supplemental Figure S7. Distribution of capillary diameters does not change with neurotoxicity.** We determined the standard deviation (SD) of all measured capillary diameters in each animal on each imaging day. Neurotoxicity did not correlate with a change in variance of capillary diameters, one-way ANOVA with Holm-Sidak posttest.
